# Strategy for drug repurposing in fibroadipogenic replacement during muscle wasting: application to duchenne muscular dystrophy

**DOI:** 10.3389/fcell.2025.1505697

**Published:** 2025-03-26

**Authors:** Izzy Matthews, Priyanka Mehra, Xavier Suárez-Calvet, Patricia Piñol-Jurado, Dan Cox, Vellia Justian, Ana Carrasco-Rozas, Zoe Laidler, Andrew Bowey, Paul Rushton, Susana López-Fernández, Jordi Díaz-Manera, Esther Fernández-Simón

**Affiliations:** ^1^ John Walton Muscular Dystrophy Research Centre, Institute of Translational and Clinical Research, Newcastle University, Newcastle upon Tyne, United Kingdom; ^2^ Department of Comparative Biomedical Sciences, The Royal Veterinary College, London, United Kingdom; ^3^ Department of Neuromuscular Diseases Laboratory, Hospital de la Santa Creu i Sant Pau, Universitat Autònoma de Barcelona, Barcelona, Spain; ^4^ Great North Children’s Hospital, Royal Victoria Infirmary, Newcastle upon Tyne, United Kingdom; ^5^ Plastic Surgery Department, Hospital de la Santa Creu i Sant Pau, Universitat Autònoma de Barcelona, Barcelona, Spain

**Keywords:** muscle dystrophies, fibro-adipogenic progenitor cells, fibrosis, adipogenesis, sarcopenia, cachexia

## Abstract

**Background:**

Understanding the cell functionality during disease progression or drugs’ mechanism are major challenges for precision medicine. Predictive models describing biological phenotypes can be challenging to obtain, particularly in scenarios where sample availability is limited, such as in the case of rare diseases. Here we propose a new method that reproduces the fibroadipogenic expansion that occurs in muscle wasting.

**Methods:**

We used immortalized fibroadipogenic progenitor cells (FAPs) and differentiated them into fibroblasts or adipocytes. The method successfully identified FAPs cell differentiation fate using accurate measurements of changes in specific proteins, which ultimately constitute a valid cellular *in vitro* platform for drug screening. Results were confirmed using primary FAPs differentiation as well as comparison with omics data from proteomics and genomic studies.

**Results:**

Our method allowed us to screen 508 different drugs from 2 compounds libraries. Out of these 508, we identified 4 compounds that reduced fibrogenesis and adipogenesis of ≥30% of fibrogenesis and adipogenesis using immortalized cells. After selecting the optimal dose of each compound, the inhibitory effect on FAP differentiation was confirmed by using primary FAPs from healthy subjects (n = 3) and DMD patients (n = 3). The final 4 selected hits reduced fibrogenic differentiation in healthy and DMD samples. The inhibition of adipogenesis was more evident in DMD samples than healthy samples. After creating an inhibitory map of the tested drugs, we validated the signalling pathways more involved in FAPs differentiation analysing data from proteomic and genomic studies.

**Conclusion:**

We present a map of molecular targets of approved drugs that helps in predicting which therapeutic option may affect FAP differentiation. This method allows to study the potential effect of signalling circuits on FAP differentiation after drug treatment providing insights into molecular mechanism of action of muscle degeneration. The accuracy of the method is demonstrated by comparing the signal pathway activity obtained after drug treatment with proteomic and genomic data from patient-derived cells.

## Background

Muscle degeneration is a complex process that occurs in several debilitating conditions including primary myopathies such as genetic diseases like muscle dystrophies, as well as acquired myopathies like inflammatory myopathies (Wallace and McNally, 2009). Additionally, it can manifest in the context of other diseases such as cancer cachexia, chronic diseases such a liver cirrhosis, neuropathies or motor neuron disorders, and during aging (Penna et al., 2013; Sakuma and Yamaguchi, 2012). All these diseases share the common characteristic of loss of strength and impaired muscle performance (Evans, 2010). Primary myopathies, such as muscular dystrophies are characterized by loss of muscle fibers leading to progressive weakness and muscle atrophy (Cassandrini et al., 2017). Sarcopenia is defined as the age-related loss of muscle mass and strength that exacerbates functional decline in the elderly population (Argilés et al., 2015). Cancer cachexia is a multifactorial syndrome that leads to muscle wasting and weakness significantly impacting the prognosis and quality of life of patients (Tisdale, 2009). Although these conditions have different causes, they share commonalities such as muscle fiber loss and atrophy, impaired muscle regeneration and activation of muscle resident cells such as fibroadipogenic progenitor cells (FAPs) (Evans, 2010). While the underlying mechanisms driving muscle degeneration in these diseases are only partially known, a complete understanding is crucial for the development of effective therapeutic interventions. Skeletal muscle has an extraordinary capacity to regenerate after an acute muscle damage. It is well known that an acute injury triggers a series of cellular and molecular consequences involving multiple cell types that cooperate to restore and preserve muscle homeostasis (Almada and Wagers, 2016; Tajbakhsh, 2009). Some of the cell types involved in this process include satellite cells (SC), FAPs, lymphocytes and macrophages that interact among them and with the extracellular matrix, not only during muscle homeostasis but also after injury (Matecki et al., 2004; Joe et al., 2010). This local interaction is usually known as the regenerative niche and is crucial for complete muscle regeneration. However, this intricate intercellular communication system declines its regenerative capacity with aging and fails in patients affected by primary myopathies such as muscular dystrophies (Cardone et al., 2023). In recent years, FAPs have been identified as key players in both muscle homeostasis and regeneration by transiently supporting the activation and differentiation of SCs (Tucciarone et al., 2018; Uezumi et al., 2011). FAPs are muscle interstitial cells that not only support muscle regeneration, but also have the potential to differentiate into adipocytes and fibroblasts (Cardone et al., 2023). In the context of diseased tissue, FAPs differentiation contributes to expansion of fibrotic and adipogenic tissue which substitutes lost muscle fibers, thereby altering muscle architecture (Relaix and Zammit, 2012; Uezumi et al., 2014). Although the majority of studies addressing the role of FAPs on muscle are performed in the context of muscle dystrophies, there are some evidences suggesting that they also have a prominent role in the pathogenesis of non-primary myopathies. For example, Liu et al. found increased predominance of FAPs and exhaustion of myogenic cells in skeletal muscle on sarcopenic conditions. Moreover, FAPs harbouring mutations in the lamin A gene, responsible for Hutchinson-Gilford progeria syndrome (HGPS) an autosomal disease associated with premature aging, undergo cellular senescence, and impair the function of muscle progenitor cells through a paracrine effect (Liu et al., 2023). In the context of patients with cancer cachexia, the increase of FAP content with exacerbated fibrotic tissue and lipids deposits was described by Judge et al. (2020). Moreover, Mallard et al. observed FAPs accumulation in skeletal muscle of patients with breast cancer and linked that increase to an increase in muscle adipose tissue (Mallard et al., 2022). Although FAPs are known to drive disease progression in these disorders, the molecular mechanisms promoting their alteration remain to be fully understood. Understanding the specific mechanisms involved in FAP differentiation can be useful to create a signalling map to find potential treatment that interferes with that differentiation.

Several research laboratories, including ours, have been using muscle-derived cells, SCs and FAPs, obtained from human samples to study the molecular mechanisms of different muscle diseases as well as to identify new potential drugs that could be useful for these conditions. However, obtaining human muscle cells can be challenging due to the invasiveness of the procedure to obtain muscle biopsies. Furthermore, the number of muscle biopsies performed for diagnosis has declined in recent years, coinciding with the popularization of next-generation sequencing techniques for diagnosis. We have developed a method to isolate human SCs and FAPs from muscle biopsies obtained for diagnosis purposes and frozen in specific conditions. Human FAPs isolated through this method retain their multipotency and can differentiate effectively into fibrogenic and adipogenic cells *in vitro*, allowing the study of molecular mechanisms involved in disease progression (Suárez-Calvet et al., 2021). Moreover, FAPs can also be immortalized maintaining their capacity to differentiate *in vitro*, thus addressing the limitation of available human muscle samples for continuous FAP isolation.

Building on our knowledge of isolating and modulating FAPs differentiation *in vitro*, we have established a cellular platform to test molecular signals that can drive FAPs fate *in vitro*, but also useful to perform screening of drug libraries that can block fibro-adipogenic differentiation and identify new therapeutic approaches for primary or secondary myopathies. Here we present a map of molecular targets of approved drugs that helps in predicting which therapeutic option may affect FAP differentiation.

## Methods

### Patients and muscle samples

Muscle biopsies were obtained from patients with a genetic diagnosis of DMD and gender matched healthy controls. Clinical data of the patients are described in Table 1.

**TABLE 1:** Clinical and genetic data of DMD patients and control included in the study.

Patient	Age at muscle biopsy	Biopsy site	Variant in the *DMD* gene	Ambulatory Status at the time of muscle biopsy	Glucocorticoid status
Samples used for scRNAseq study					
Healthy control 1	12	Quadriceps	Not applicable	Ambulant	No
Healthy control 2	10	Gracilis	Not applicable	Ambulant	No
DMD 1	10	Biceps brachii	Deletion 49-52	Ambulant	Yes
DMD 2	9	Biceps brachii	Deletion 18-44	Ambulant	Yes
DMD 3	9	Biceps brachii	Deletion 45–52	Ambulant	Yes
Samples used for proteomic study					
Healthy control 1	10	Biceps brachii	Not applicable	Ambulant	No
Healthy control 2	22	Biceps brachii	Not applicable	Ambulant	No
Healthy control 3	14	Biceps brachii	Not applicable	Ambulant	No
DMD 1	9	Biceps brachii	Deletion 48-52	Ambulant	Yes
DMD 2	9	Biceps brachii	Deletion 50-52	Ambulant	Yes
DMD 3	7	Biceps brachii	Deletion 52	Ambulant	Yes

We obtained informed consent forms signed by parents or legal guardians as well as patient’s assent forms for donating these samples to research. Muscle samples from the biceps brachii of age- and gender-matched controls were obtained by the orthopaedic surgery department of Hospital de la Santa Creu I Sant Pau in Barcelona. Primary cells were obtained from the John Walton Muscular Dystrophy Research Centre Biobank (REC Ref: 19/NE/0028), which is supported by the NIHR Newcastle Biomedical Research Centre. The study was approved by the Ethics Committee of the Hospital de la Santa Creu I Sant Pau Hospital.

### Cell culture

Frozen muscle explants from healthy controls and DMD patients were cultured to obtain FAPs as previously described (Suárez-Calvet et al., 2021). Briefly, muscle fragments were cultured in gelatine-plasma coated dishes with FAP medium (DMEM-GlutaMAX (ThermoFisher [Gibco], Waltham, MA) supplemented with 20% FBS (Gibco), 1%PS (Lonza, Basilea, Switzerland) and 2.5 ng/mL of basic fibroblast growth factor (Peprotech, Rocky Hill, NJ)). Sprouting cells from explants were trypsinized (Gibco), expanded and subcultured in Cell + culture flasks (Sarstedt, Nümbrecht, Germany).

### Cell sorting

Cultured human muscle derived cells were labelled using anti‐PDGFRα (BAF322, R&D Systems, Minneapolis, MN) followed by streptavidine‐PECy5 (405202, Biolegend, San Diego, CA) and anti‐CD56 (Milteny-Biotech). Stained cells were sorted on a FACSAria using FACSDiva software (Becton Dickinson, Ashland, OR). Doublet cells were excluded using forward scatter area and height and fluorescence minus one (FMO) controls were used to determine positivity. PDGFRα+/CD56− fraction was defined as FAPs. Human FAPs isolated from healthy human sample were transduced with a retroviral vector containing a sequence encoding the catalytic subunit of human telomerase reverse transcriptase (hTERT) alone or with both hTERT and cyclin-dependent kinase 4 (Cdk4) as described previously.

### *In vitro* differentiation and pharmacological treatment

FAPs were seeded at 5,000 cells/cm^2^ concentration in 96 well plates in FAP growth medium. To achieve adipogenic differentiation, media was changed into adipogenic differentiation medium (StemPro, Gibco) for 6 days. In order to induce fibrogenic differentiation, FAPs were cultured for 3 days with DMEM-GlutaMAX with 10% FBS with 5 ng/mL TGFβ (R&D Systems). Pharmacological compounds were obtained from the Wnt/Hedgehog/Notch Compound Library (186 compounds) and Tyrosin-kinase inhibitors (TKI) Library (322 compounds) from MedChemExpress, United States. Adipogenesis differentiation was initially assessed using the Wnt/Hedgehog/Notch Compound Library. Negative controls (non-differentiated FAPs) were maintained in DMEM+2% FBS and positive controls (adipogenic-differentiated FAPs) were treated with StemPro+0.01% DMSO (vehicle compound). Each compound was added to one well and incubated for 6 days to allow adipogenic differentiation process. Fibrogenic differentiation was initially assessed using the TKI library. Negative controls (non-differentiated FAPs) were maintained in DMEM+10%FBS while positive controls (fibrogenic-differentiated FAPs) were treated with fibrogenic medium+0.01%DMSO (vehicle compound). Compounds were incubated for 3 days to allow complete fibrogenic differentiation process.

### In-cell western assay

After pharmacological treatment, collagen-I expression was analysed in fibrogenic differentiation and perilipin-1 in adipogenic differentiation using quantitative in-cell western (ICW). Plates were rinsed with PBS and fixed with 4% PFA for 10 min. After washing steps and blocking with casein (ThermoFisher) solution for 30 min, goat anti-collagen I (1:500; Cat. 1310-01; Southern Biotech, Birmingham, AL) or rabbit anti-perilipin A/B (Cat. P1873–200UL; 1:500; Sigma-Aldrich, St. Louis, MO) was added overnight at 4C. After the washing steps, wells were incubated with either donkey anti-goat IRDye800 or donkey anti-mouse IRDye800 secondary antibody (1:1000; Li- COR, Lincoln, NE) and CellTag IRDye 700 (Li-COR) for 1 h. After washing with PBS, the fluorescent signal was measured by an Odyssey Imaging system (Li-COR) and normalized by cell number measured in the 700 nm channel. To analyse the % of inhibition by each drug, each condition was normalized to the positive control (fibrogenic or adipogenic differentiated FAPs). After that, we represented each drug into a map were the 0% represented the standard fibrogenic or adipogenic differentiation of FAPs and the effect of each compound was plotted in the negative side of the graph while drugs promoting the differentiation are represented in the positive side of the graph. At this stage, drugs were tested at n = 1.

### IC50 and cell viability assay

FAPs were plated in 96-well plate at 5,000 cell/cm^2^ density for 24 h. Then, cells were treated with the pre-selected compounds diluted in adipogenic or fibrogenic differentiation media in 12 descending concentrations (2,200–3,124 nM) and the vehicle. The differentiation performed at this step was according to the drug original plate (fibrogenic differentiation for TKI library or adipogenic differentiation for Wnt/Hedgehog/Notch Compound Library and compounds were tested in triplicates (n = 3). After differentiation process, FAPs were stained with PrestoBlue Cell Viability Reagent (Invitrogen) following manufacturer’s instructions. Fluorescence was measured at wave-lengths 570 nm excitation and 600 nm emission using a microplate reader (Varioskan LUX, ThermoFisher). After that, FAPs were rinsed with PBS and the ICW protocol was performed in the same plate to stain collagen-I and perilipin proteins.

### Mechanistic model of cell functionality

The normalized gene or protein expression data obtained from single-cell RNA-seq (Fernández-Simón et al., 2024) (data available at https://singlecell.broadinstitute.org/single_cell/studies/66703832771a5b0208cf92e3) and proteomic dataset was used to predict which signalling circuits were differentially expressed in the DMD condition compared to the control condition. Quantitative proteomics was performed using a Lumos Orbitrap mass spectrometer (Thermo Fisher Scientific,San Jose, CA, United States) coupled to an EASY-nLC 1000 (ThermoFisher Scientific (Proxeon), Odense, Denmark). Samples were analysed with the MaxQuant software (version1.6.1.0) through the human Swissprot database. Single cell RNA-seq from FAPs were partitioned into Gel Bead-In-Emulsions (GEMs) by using the Chromium Controller system (10X Genomics). cDNA sequencing libraries were prepared using the Next GEM Single Cell 3′ Reagent Kits v3.1 (10X Genomics, PN-1000268), following manufacturer’s instructions. cDNA libraries were indexed by PCR using the PN-220103 Chromiumi7 Sample Index Plate. Size distribution and concentration of 3′cDNA libraries were verified on an Agilent Bioanalyser High Sensitivity chip (Agilent Technologies). Finally, sequencing of cDNA libraries was carried out on an Illumina NovaSeq 6,000 using the following sequencing conditions: 28 bp (Read 1) + 8 bp (i7 index) + 0 bp (i5 index) + 89 bp (Read 2), to obtain approximately 20–30.000 reads per cell. We checked the quality of the cells in each sample in R (version 4.2.1). For downstream analysis, cells with less than 1,000 reads or 500 genes and more than 5% mitochondrial reads were filtered out (Wolock et al., 2019). Using DoubletFinder (version 2.0.3), we identified and filtered out the doublets from each sample. 2000 highly variable genes were then identified using which we conducted principal component reduction and clustering of the data.

### Bioinformatic analysis

To reveal the precise biological properties of each conditions, we used Metascape (http://metascape.org) to perform enrichment analysis including KEGG Pathway, GO Biological Processes, Reactome Gene Sets, Canonical Pathways, CORUM, WikiPathways and PANTER Pathway. Genes with a log2FC > 0.5 were analysed for each condition.

The HiPathia method was used to estimate signalling circuit activities within DMD condition from the corresponding normalized gene and protein expression values. HiPathia identifies signalling circuits using nodes that represent genes or protein and edges that represents the interaction. The software estimates the signal transduction along these pathways by considering both the structure of the pathway and gene expression levels. We initially excluded circuits that maintained consistent activation statuses across the studied conditions. Then, we selected the circuits that were represented by the pathways targeted by the drugs used. Results from the scRNAseq and proteomic studies were normalized using the log-fold change (logFC) of each protein comparing the expression of the DMD condition against the healthy control condition. The accuracy of the classification was assessed based on the log fold change (logFC) of each circuit and averaging the total circuits by pathway. The HiPathia method uses a Wilcoxon test was used to assess differences in pathway activity between controls and DMD samples (Fernández-Simón et al., 2024).

## Statistics

Initial screening was performed on n = 1 and compounds that reduced ≥30% of the fibroadipogenic differentiation were selected. From that point, all the following experiments were performed with technical replicates (n = 3). Results are expressed as mean ± standard error of means (SEM). Differences among the groups were analysed using ANOVA Test (either 1-way or 2-way as indicated in the figure legends). When ANOVA revealed significant differences, the Tukey *post hoc* test was performed. In case the ANOVA test was not applicable, the non-parametric Mann-Whitney U test was used. The significance level was set at P < 0.05. Statistical analyses and graphic representations were performed with GraphPad Prism Software 8 (La Jolla, CA, United States). When using HiPathia software, the statistical test to assess differences consisted of Wilcoxon test.

## Results

### Building a FAPs differentiation response map

**FIGURE 1:**
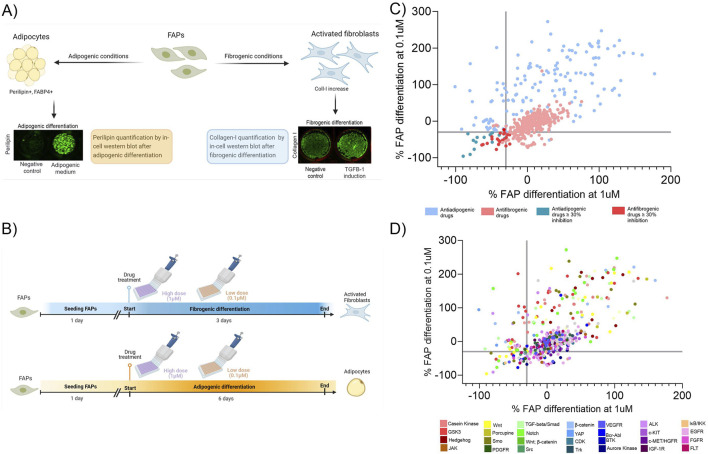
Overview of the *in vitro* platform. **(A)** FAPs cultured in two different media (fibrogenic or adipogenic conditions) were analysed for the fibrogenic or adipogenic differentiation by quantifying the expression of collagen-1 or perilipin-1, by in-cell western, respectively. **(B)** Timeline representation of fibrogenic and adipogenic differentiation using the drug libraries. Each drug was added at a high and low dose at the same time but in different wells. **(C)** Map response graph showing the effect of the drug libraries after treatment at high (1 µM) and low (0.1 µM) concentrations. Adipogenesis and fibrogenesis inhibition are both shown on the same graph and the axes represent the percentage of the fibrogenic or adipogenic inhibition at a high and low dose. **(D)** Target effect map based on the specificity of each compound on inhibiting or promoting fibroadipogenic differentiation. The axes represent the percentage of the fibrogenic or adipogenic inhibition at a high and low dose.

To reproduce the fibrogenic and adipogenic differentiation of FAPs, we differentiated immortalized FAPs into fibroblasts and adipocytes *in vitro*. As a readout, we quantified collagen-I and perilipin-I expression using in-cell Western blot (ICW) technique (Figure 1A). With that method, we assessed how this differentiation is influenced by two different drug library containing multiple compounds that could potentially inhibit fibrogenesis and/or adipogenesis. For adipogenic differentiation, we used a library of compounds interfering with the Wnt/Hedgehog/Notch pathways as these have been identified to be involved in adipogenesis (Marinkovic et al., 2019; Keats et al., 2014; Reggio et al., 2020). Regarding fibrogenesis, we used a library of 339 tyrosin-kinase inhibitors (TKI) since they been shown to influence fibrogenesis (Piñol-Jurado et al., 2018; Zhang et al., 2016). To analyse the effect of the libraries, drugs were added at a high (1uM) and low (0.1uM) concentration (Figure 1B) and differentiated into fibroblasts using the TKI library or adipocytes using the Wnt/Hedgehog/Notch Compound Library. Negative controls (containing the same vehicle as drugs) were also added to the plates and served as a reference for the standard differentiation rate enabling the calculation of the induction or inhibition of each compound used. Specifically, drugs were defined as a hit when the adipogenic or fibrogenic differentiation potential was decreased by more than 30% at both high and low doses compared to the negative control. We then plotted the inhibition of fibrogenesis by the TKI inhibitors and adipogenesis by the Wnt/Hedgehog/Notch interfering compounds at both doses on the same graph (Figure 1C) and identified a total of 29 drugs interfering with FAPs differentiation (Table 2). Based on the mechanism of action of each drug we constructed an inhibitory map by selecting the main target of each compound (Figure 1D).

**TABLE 2:** Pre-selected compounds identified in the first screening. Information about FDA status, muscle related studies, *in vivo* validation and clinical trial status are included.

Name	FDA status	Muscle-related studies	*In vivo* validation	Human trials status
Ponatinib	Approved	No	Yes (Zhang et al., 2016)	Active (NCT06813079)
Dacomatinib	Approved	No	Yes (Xu et al., 2021)	Active (NCT06486142)
WS3	Not approved	No	No	No
TG10129	Not approved	No	Yes (Hidalgo et al., 2016)	No
Geldanamycin	Not approved	Yes (Esteban-Medina et al., 2019)	Yes (Sakuma, 2017)	Completed (NCT01613950)
GSK1838705	Not approved	No	No	No
PD173074	Not approved	Yes (Zhuang and Liu, 2014)	Yes (Bowen et al., 2015)	No
CC223	Not approved	No	Yes	Completed (NCT01896323)
ALW-II-21-27	Not approved	No	No	No
ACTB1003	Approved	No	No	Completed (NCT03583125)
AD80	Not approved	No	No	No
Sonidegib	Approved	No	Yes (Contreras et al., 2016)	Recruiting (NCT05669339)
Gigantol	Not approved	No	No	No
β-catenin/CBP-IN-1	Not approved	No	Yes (Wang et al., 2023)	No
Bruceine D	Not approved	Yes (Fiore et al., 2016)	yes (Mu et al., 2020)	No
CB-103	Not approved	No	Yes (Liu et al., 2024)	Active (NCT05774899)
Salinomycin	Not approved	No	Yes (Fernández-Simón et al., 2022)	No
Triptonide	Not approved	Yes	No	No
GSK-3 inhibitor1	Not approved		Yes	No
L-Quebranchitol	Not approved	No	Yes (Suárez-Calvet et al., 2023)	No
AZD 1080	Not approved	No	Yes (Schüler et al., 2021)	Discontinued
RGF 286638-free base	Not approved	No	Yes (Weber et al., 2022)	No
Advavint	Not approved	No	Yes (Conboy et al., 2003)	Completed (NCT05603754)
RGF286638	Not approved	No	Yes (Weber et al., 2022)	No
SAG	Not approved	No	Yes (Van Der Biessen et al., 2014)	No
CCT251545	Not approved	No	Yes (Mallard et al., 2022)	No
Halofuginone	Not approved	Yes (Cirstea et al., 2013)	Yes (Cirstea et al., 2013)	Terminated (NCT02525302)
KYA1797K	Not approved	No	Yes (Yang et al., 2020)	No
LH846	Not approved	No	No	No

### Characterization of selected drugs

**FIGURE 2:**
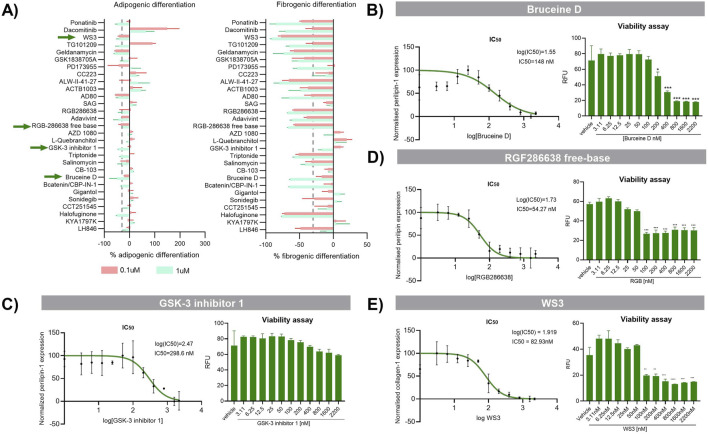
Characterization of the preselected drugs. **(A)** The pre-selected drugs that did not showed a decrease at the total amount of cell underwent another round of screening for adipogenic and fibrogenic differentiation with technical triplicates being employed. At the end of the differentiation, the percentage of inhibition of each drug at high and low dose was analysed. The X-axis represent the percentage of inhibition measured for the fibrogenic differentiation and the adipogenic differentiation. **(B)** Bruceine D, **(C)** GSK-3 Inhibitor 1, **(D)** RGF286638 free-base and **(E)** WS3 compounds were analysed for viability assay using a range of decreasing concentrations starting from the 2200 nM−3.11 nM. RFU; Relative fluorescence units. Following viability assay, fibrogenic or adipogenic differentiation was analysed using ICW. Data is represented as the mean of three replicates ±standard error of the mean. Results were statistically analysed using one-way ANOVA, followed by Tukey *post hoc* test. Statistical significance was set at P < 0.05. **P < 0.01; ***P < 0.001.

To further characterize the selected drugs, we ensured that the inhibition of fibrogenesis with the TKI did not promote adipogenesis and similarly, that the inhibition of adipogenesis with the Wnt/Hedgehog/Notch compounds did not promote fibrogenesis. We used triplicates and put them in either an adipogenic or fibrogenic differentiation media as in our previous assay. At this stage, drugs showing an increase in fibrogenesis or adipogenesis were eliminated (Figure 2A) as well as those that reduced the total amount of cells (measured by CellTag). Out of the 29 compounds, 8 compounds reduced fibrogenesis and adipogenesis at ≥30% but 4 were eliminated since they showed reduced number of cells (Salinomycin, Adavivint, AD80 and Geldanamycin). Final compounds selected for the next step were: Bruceine D, GSK-3 inhibitor 1, RGF 286638-free base. To find the most effective and non-cytotoxic dose, the pre-selected drugs were analysed for fibrogenic or adipogenic differentiation together with a viability assay using a range of decreasing concentrations (from 22 μM to 3.11 nM) (Figures 2B–D). Adjustments to find the exact concentration that had an effect on FAPs without affecting viability were performed in primary cell line of FAPs isolated from a healthy subject (Supplemental Figure 1). Out of the initial 508 drugs screened, we narrowed down the list to a final 4 compounds.

### Mechanistic model of cell signalling

Once we established the *in vitro* system, we aimed to further validate the signalling pathways involved in the degenerative process of FAPs. To validate this system, we analysed whether the upregulated pathways observed in FAPs isolated from pathological conditions were decreased after drug treatment in our *in vitro* assay.

**FIGURE 3:**
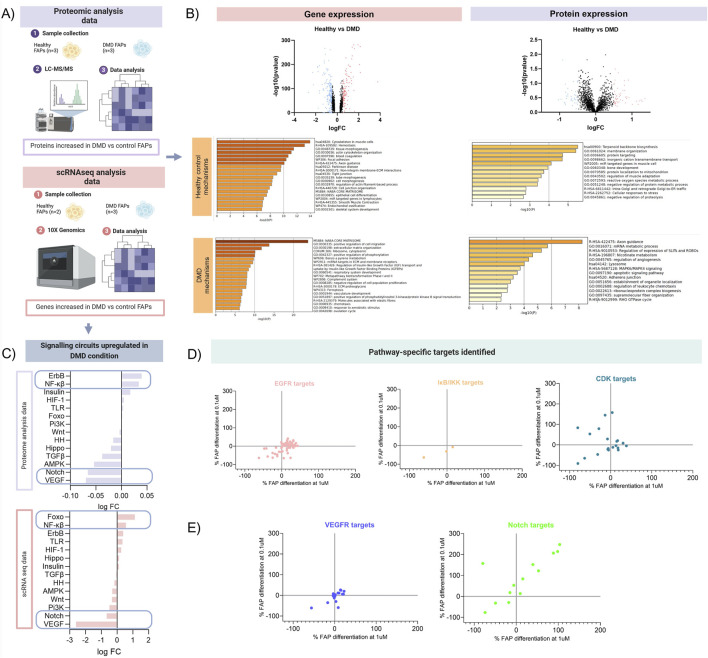
Schematic overview of the mechanistic model analysis. **(A)** Analysis of proteomic and transcriptomic data from DMD FAPs. Datasets from scRNAseq data from 2 healthy controls and 3 DMD patients and datasets from proteomic data from 3 healthy controls and 3 DMD patients ware used for this study **(B)** Volcano plots showing the differences at the genomic and proteomic level. Genes and proteins with a log2FC > 0.5 were analysed for each condition. **(C)** Signalling circuits upregulated in DMD condition were analysed using the proteomic and scRNAseq data. HiPathia software was used to reveal which signalling circuits were upregulated in the DMD condition. **(D)** Target-specific map of the most upregulated pathways identified in the proteomic and transcriptomic analysis. Drugs used in the 2 libraries previously described that targeted the pathways more present in the DMD condition (EGFR, NF-Kβ and CDK) were plotted individually. **(E)** Target-specific map of the 2 most downregulated pathways identified in the proteomic and transcriptomic analysis. Drugs used in the 2 libraries previously described that targeted the pathways less present in the DMD condition (VEGFR, Notch) were plotted.

We decided to examine deregulated pathways in FAPs isolated from Duchenne muscular dystrophy patients, a genetic disorder characterized by muscle degeneration. DMD is one of the most severe forms of muscular dystrophies leading to rapid degeneration of the skeletal muscle accompanied by an increase in fibrotic and adipogenic tissue. For this reason, analysing deregulated pathways in FAPs isolated from DMD patients could be a valuable strategy for studying the molecular mechanisms involved in the process of muscle degeneration. By combining results from cell transcriptomic and proteomic studies, we unravelled the underlying mechanisms of cell signalling in FAPs, providing a functional link between gene and protein-level data and disease-related cellular mechanism of interest. On the one hand, we performed single-cell RNA sequencing (scRNAseq) study using 10X Genomics and Illumina technology to analyse the gene signatures of FAPs isolated from healthy samples (n = 2) and DMD patients (n = 3) (Xu et al., 2021) (doi: 10.3389/fcell.2024.1399319). In parallel, we performed mass spectrometry to analyse the protein expression of FAPs isolated from healthy samples (n = 3) and DMD patients (n = 3) (Figure 3A). To obtain a comprehensive understanding of the variations in gene and protein expression between DMD and control FAPs, we first analysed the differences at the genomic and proteomic level comparing the healthy samples and the DMD samples. Differences in proteins and genes are represented in a Volcano plot in Figure 3B. To reveal the precise biological properties of each condition, we used Metascape. Genes and proteins with a log_2_FC > 0.5 were analysed for each condition. Top genes upregulated in the healthy condition (blue dots) were involved with the actin cytoskeleton organization and ECM interaction as well as skeletal system development while top genes in the DMD condition (red dots) were involved with cell migration, regulation of insulin-like growth factor or the complement system. Top proteins upregulated in the healthy condition were involved in cell homeostasis such as protein localization between organelles in the cell, membrane organization or regulation of muscle adaptation. However, upregulated proteins in the DMD condition were involved with mRNA metabolic process, regulation of angiogenesis or adherent’s junctions.

Then, we analysed potential changes in signalling pathways using high throughput pathway interpretation and analysis (HiPathia) tool. This tool transforms uninformative gene expression data into signalling pathway circuit activities (Hidalgo et al., 2016; Esteban-Medina et al., 2019). The signalling circuits selected are those that exhibit the most significant change in activity either upregulated or downregulated pathways (Figure 3C). The increased cell signalling circuits identified in both datasets allowed us to estimate the signalling activity profiles upregulated in pathogenic conditions. Upon comparing the two studies, we observed that signalling circuits related to Forkhead box O (FOXO), Nf-kB or ErbB pathways were upregulated in DMD FAPs, while circuits related to VEGF and Notch were decreased in DMD FAPs.

This orthogonal integrative analysis enabled us to predict the mechanism of action of drugs and to validate whether pathways increased in the DMD condition *in vitro* were in accordance with the highest inhibited targets. For instance, the ErbB signalling, which is associated with the epidermal growth factor receptor (EGFR), was found to be one of the molecular pathways most upregulated in DMD FAPs. When we analysed how FAPs respond to EGFR inhibitors, we observed that most of these compounds decreased FAP differentiation, although the pattern of inhibition was not completely homogeneous. Another example of the most representative pathways is the Nf-kB signalling pathway. When we analysed which of our compounds targeted that pathway, we observed that three compounds inhibited the IκB kinase, which is part of the upstream Nf-KB signal transduction cascade. All these three compounds showed an inhibitory pattern in FAPs differentiation. Finally, we analysed the effect of inhibiting insulin or FOXO pathway. Since FOXO is a transcription factor, we did not identify any drug targeting the factor directly. However, HiPathia tool classified-FOXO signalling by upregulation of cyclin kinase proteins. Once we analysed the inhibition of different CDK isoforms, we observed that most of them presented a negative regulation of FAPs differentiation (Figure 3D). That observation shows that hallmarks playing some type of upstream regulator role in pathogenic conditions are the ones most responsive to inhibitory treatments.

Conversely, we observed that VEGF and Notch signalling pathways were downregulated in the DMD FAPs *in vitro*. Targets of these pathways did not show an inhibitory effect, suggesting that molecular pathways that are not physiologically upregulated in DMD may not possess any regulatory activity on FAPs differentiation. We observed that targeting Notch pathway resulted in an increase of both fibro/adipogenic differentiation, although some of these drugs reduced the differentiation potential of FAPs. This heterogeneous response to the same pathway might be associated with the suppressive mechanism of adipogenesis related to Notch signalling. It has been previously studied that Notch reduces adipogenesis (Marinkovic et al., 2019), and therefore Notch inhibitors could lead to an increased differentiation of FAPs into adipocytes (Figure 3E).

**FIGURE 4:**
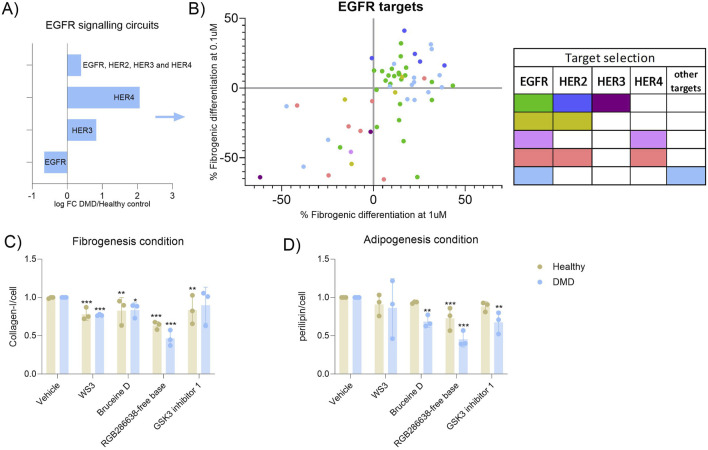
Analysis of specific receptors from the EGFR pathway. **(A)** Bar graph showing the expression of the different EGFR receptor expression observed in the scRNAseq dataset when the DMD condition was analaysed against the healthy control condition. **(B)** Dot-plot showing the specificity of each drug targeting the EGFR pathway against each receptor of the EGFR pathway. Green dots correspond to compounds that only target the EGFR receptor, dark blue dots correspond to compounds that only target the HER2 receptor, purple dots correspond to compounds that only target the HER3 receptor, olive dots correspond to compounds that target the EGFR and HER2 receptors, violet dots correspond to compounds that target the EGFR and HER4 receptors, salmon dots correspond to compounds that only target the EGFR, HER2 and HER4 receptors and light blue dots correspond to compounds that only the EGFR and other receptors that are not EGFR. **(C)** Validation of the best candidates selected in primary FAPs from three healthy aged-matched controls and three DMD patients. Fibrogenesis inhibition was of; −22%, pvalue <0.0001, −36%, pvalue <0.0001, −18%, pvalue = 0.0028 and −17%, pvalue = 0.0049 in healthy samples while in DMD samples the reduction was of; −24% pvalue = 0.006, −54%, pvalue <0.0001, −17%, pvalue = 0.018 and −11%, pvalue = 0.275. **(D)** The inhibition of adipogenesis consisted of; −9%, pvalue = 0.776, −28%, pvalue = 0.038, −7%, pvalue = 0.914 and −12%, pvalue = 0.580 in healthy FAPs while in DMD samples, the reduction was of; −14%, pvalue = 0.396, −55%, pvalue <0.0001, −32%, pvalue = 0.0017 and −33%, pvalue = 0.0012. Results were statistically analysed using two-way ANOVA followed by Tukey *post hoc* test. Statistical significance was set at P < 0.05. **P < 0.01; ***P < 0.001.

Since the inhibitory pattern according to each pathway was not homogeneous due to some drugs targeting the same pathway could inhibit but also had no effect on FAP differentiation, we aimed to further assess the specificity of targeting single proteins from a determined pathway. We decided to focus on the EGFR pathway as it was the highest upregulated pathway in both the transcriptomic and proteomic study. The EGFR pathway has been previously linked to fibrosis (Sakuma, 2017; Zhuang and Liu, 2014) and all the compounds used in our study were from the TKI library, so we performed the first inhibition assay in fibrogenic conditions. The epidermal growth factor RTK family consists of four members: EGFR (ErbB1, HER1), ErbB2 (HER2), ErbB3 (HER3) and ErbB4 (HER4). By evaluating the circuits upregulated or downregulated in our scRNA-seq dataset, we observed that HER4 and HER3 signalling pathways were increased, while EGFR pathway was decreased. When circuits belonging to all 4 receptors were analysed, there was an increase of activity detected (Figure 4A). By analysing each target of the pathway in our library response map (Figure 4B), we observed that while EGFR and HER2 inhibitors did not influence FAP differentiation (green and dark blue dots, respectively), drugs targeting HER3 specifically inhibited FAP fibrogenic differentiation (purple dots). We could not analyse the effect of only inhibiting HER4 because none of the drugs of the drugs used in our library specifically target that receptor. However, we identified one compound interfering with HER4 and EGFR that inhibited FAPs differentiation (violet dot). Most of the compounds targeting HER4, HER2 and EGFR did also reduce FAPs differentiation (red colour) suggesting that HER3 and HER4 signalling may be more involved in fibrofatty differentiation than HER2 and EGFR (Figure 4B). This is another example of how our *in vitro* platform can help in the analysis of the molecular mechanisms that are altered in pathogenic conditions and which type of drugs can be developed to inhibit fibrofatty expansion in muscle tissue.

### Validation of selected drugs in primary cells

Finally, we wanted to assess whether the selected drugs identified in our assay had a similar effect on primary cells obtained from healthy individuals and from patients. We isolated FAPs from muscle tissue from three DMD patients and three healthy aged-matched individuals. By using the differentiation assay as previously described, we treated FAPs with the four top candidates of our libraries. Despite the high variability observed, which is probably related to the inherent variability of using primary cells from different individuals, we observed that the four candidate drugs reduced fibrogenesis and/or adipogenesis. In general, the inhibition was higher when samples from DMD patients were used, suggesting that FAPs isolated from a perturbed microenvironment could respond more to antifibrotic and antiadipogenic treatment and supported our previous results showing that the molecular pathways previously identified could be also upregulated in these cells (Figures 4C, D).

## Discussion

Hereby we have described a cellular platform for the study of the molecular mechanisms involved in muscle degeneration. Due to the lack of available tissue from specific conditions such as muscular dystrophies, sarcopenia or cachexia, the development of an *in vitro* cell system to study molecular mechanisms of the disease and screen libraries of drugs is key to progress in the identification of new therapies for these diseases. Our findings demonstrate that human FAP cells are effective for studying fibrogenesis and adipogenesis, making them valuable for testing drug efficacy.

Muscle degeneration, involving the gradual loss of muscle tissue and function, is central to conditions like muscular dystrophies, sarcopenia, and cancer cachexia. Understanding its mechanisms is crucial for developing treatments, but effective therapies remain elusive, focusing mainly on symptom management and supportive care (Sakuma and Yamaguchi, 2012; Bowen et al., 2015). Among the different cells involved in muscle degeneration, FAPs play a key role in muscle degeneration due to their capability of differentiating into fibrotic and adipogenic lineages in response to injury or disease (Contreras et al., 2016; Wang et al., 2023). Dysregulated FAPs lead to fibrotic tissue deposition and adipocyte infiltration, exacerbating tissue degeneration and impairing regeneration capacity (Fiore et al., 2016). The complexity of the molecular pathways, patient heterogeneity, and sample collection challenges hinder therapy development. Despite limited knowledge about the mechanisms of FAP differentiation in muscle dystrophies, sarcopenia, and cancer cachexia, studies suggest common pathways are activated in FAP regulation. An example of this can be the RhoA/ROCK signalling in regulating the cytoskeletal dynamics and fibrogenic differentiation. Mu et al. studied the role of RhoA/ROCK in Hutchinson-Gilford progeria syndrome (HGPS) and observed that RhoA/ROCK was activated in FAPs from patients and from the Zmpste24−/− murine model of HGPS, promoting and increasing the cytoskeletal stiffness. By inhibiting RhoA/ROCK activation, cytoskeletal stiffness was rescued in skeletal muscle promoting muscle regeneration (Mu et al., 2020). On the other hand, RhoA/ROCK pathway activated by lactate was studied in cancer cachexia as a contributor of adipose catabolism and muscle wasting. Inhibitors of ROCK showed no influence in the tumour growth on animal models but alleviated tissue weight loss in mice (Liu et al., 2024). The same pathway was increasingly detected in DMD samples from patients promoting activation of FAPs. Treating a D2-mdx murine model of DMD mice model with the ROCK inhibitor fasudil improved muscle function and reduced expression of markers of fibrosis in muscle (Fernández-Simón et al., 2022).

Given these premises, establishing a cellular platform with immortalized human FAPs capable of differentiating into fibroblasts and adipocytes could be valuable for studying the molecular mechanisms behind fibrofatty degeneration in muscle across different pathological conditions leading to the identification of potential molecular druggable candidates. Our methodology provides a versatile framework applicable to various conditions involving fibrosis and adipogenesis. Mapping drug responses within specific signalling pathways provides a structured view of FAP differentiation in response to fibrogenic and adipogenic stimuli.

The *in vitro* approach described here links omics data with signalling circuits responsible for fibro-adipogenic differentiation, demonstrating that FAPs mimic some of the pathological changes observed *in vivo* in DMD patients and, therefore they are a suitable cell model for research. Additionally, we validated the efficacy of our system by testing the four best candidate drugs in primary cells from both healthy individuals and DMD patients. Our results showed increased signalling of the EGFR, NFKB and FOXO pathway in DMD conditions. Although the role of EGFR on FAPs has just recently been shown to be upregulated in transcriptomic analysis from DMD patients compared to healthy patients (Suárez-Calvet et al., 2023), more studies have been focused on its role on secondary muscle diseases. The perturbation of EGFR signalling through aging (Schüler et al., 2021) and the amelioration of cancer cachexia using EGFR inhibitors (Weber et al., 2022) are examples of the importance of EGFR pathway in secondary muscle diseases. Our results support a role of EGFR pathway in FAPs differentiation as the use of EGFR inhibitors reduced fibrotic differentiation potential of FAPs. On the other hand, Notch and VEGFR pathways were downregulated in the transcriptomic and proteomic data obtained from DMD patients. However, the response of drugs targeting Notch did not present a homogeneous pattern of inhibition in our cell platform. This heterogeneous response to inhibitors of the same pathway may be associated with the suppressive mechanism of adipogenesis related to Notch signalling pathway. Previous studies found that Notch negatively controls adipogenesis (Liu et al., 2023). The downregulation of Notch signalling in pathologic conditions has also been reported by Marinkovic et al., who observed a decreased activity of Notch in DMD mice model. Moreover, Conboy et al. reported a reduced activation of Notch in aged mice (Conboy et al., 2003). We believe that this suppressive mechanism already reported was the main cause of observing a heterogeneous pattern of FAP response in our map, where some drugs did supress FAP differentiation while others seems to increase that differentiation. The final compounds selected in our study target most of the pathways that we have found to be increased in the DMD condition. RGF-286638 has been previously tested in animal model in the non-free base formula (Van Der Biessen et al., 2014) and it is a multigarget protein kinase inhibitor that has shown activity against Cyclin-dependent kinases, JAK2, MEK1 or GSK-3β (Cirstea et al., 2013). Bruceine D has shown to suppress oxidative stress and inflammatory response in a mice model of Parkinson’s disease as well as inhibiting growth and metastasis of tumour in mice (Yang et al., 2020). It also targets different pathways such as MAPK, JAK/STAT or Notch, signalling (Tan et al., 2019). On the other hand, GSK-3 inhibitor 1 has been previously identified as an inhibitor of adipogenesis in FAPs (Marinkovic et al., 2019). WS3 is the most potent diarylurea analogue that has previously shown activity as a tumour suppressor and which main target is Erb3 and IKK (Shen et al., 2013). Diarylureas are tyrosin-kinase inhibitors that are used as anticancer agents but also as antiviral or anti-inflammatory (Catalano et al., 2021).

The main limitation of our protocol is that drugs targeting a specific protein often exhibit the phenomenon of promiscuity, meaning that they interact with unintended secondary targets. This could explain why our molecular signalling target map is not always consistent showing that drugs targeting the same pathway can have either an increase or a decrease of FAP differentiation potential. An example of that are the targets of Bruceine D and RGF-286638 free base, since both are multitarget protein kinase inhibitors. Elucidating these interactions could be explored using our *in vitro* cell platform, with the aim of minimizing adverse effects and enhancing drug discovery. By screening additional compounds, we can further link the relationship between compound structure and mechanism of action.

Another limitation of our work is that is based on immortalized FAPs. Although these cells are directly obtained from human samples and are very useful for large screening assays, they don’t exhibit the same functional properties as primary cells. The immortalization process can result in changes in the gene expression profile, metabolic pathways that regulate cellular function or the release of ECM factors to the media, which are important mechanisms occurring in muscle tissue during the progression of the disease (Maqsood et al., 2013; Voloshin et al., 2023). These discrepancies can influence the efficacy of therapies targeting fibrosis or adipogenesis. For that reason, we wanted to validate the effect of the best compounds on primary FAPs, which have not been modified genetically and are more reliable models for preclinical testing of drug candidates. The ability of primary FAPs to respond to external treatment make them more representative of what is occurring *in vivo*.

We believe that studying shared molecular hallmarks occurring in muscle degeneration present an opportunity to analyse the involved mechanisms and to select candidate drugs. This approach will enable larger groups of patients to benefit from therapies by targeting overlapping pathological mechanisms. Further studies integrating data from other pathogenic conditions such as sarcopenia or cachexia, will be crucial for connecting the signalling pathways analysed with the phenotypic and functional effects of cells after treatment.

With the rapid advancement in omics technologies, predicting cell behaviour through genomic and proteomic information for optimal treatment strategies becomes a paramount objective in translational medicine. The ability to predict drug response by omics data can be challenging due to the lack of molecular interactions that drive cellular response *in vivo*. I*n silico* platforms can also be useful to predict potential interactions between drugs and therapeutically relevant targets, however there are some limitations. First, the translation of the results of the *in silico* analysis needs to be validated in order for the results to be reliable. Second, the lack of specific mechanistic studies. Several available platforms define cellular response to pharmacological agents but all of them are based on human cancer cell lines (Hidalgo et al., 2016; Santos et al., 2017). Therefore, understanding the underlying molecular mechanism of action in muscle derived cells by which drugs affect the increase of fibrofatty deposition constitutes a key step in understanding each drug specific cause-effect relationship.

## Conclusion

Despite advances in the understanding of the underlying mechanisms driving muscle degeneration, effective treatments remain limited. Targeted therapies aimed at preserving muscle mass and function hold promise for addressing muscle degeneration. Since FAPs have a prominent role in muscle degeneration, therapeutic approaches modulating its signalling pathways could be useful across pathogenic conditions characterized by fibroadipogenic expansion. Unravelling the intricate interplay between cellular and molecular pathways involved in muscle degeneration needs to be addressed to find new potential targets.

## Data Availability

The data presented in the study are deposited in the Single cell Broadinstitute.org repository, accession number SCP2678, available at: https://singlecell.broadinstitute.org/single_cell/study/SCP2678/single-cell-rna-sequencing-of-human-faps.

## Supplementary material

SUPPLEMENTARY FIGURE 1 Adjusted dose of selected compounds. The final selected drugs were analysed for viability assay when fibrogenic differentiation was analysed. RFU; Relative fluorescence units. **(B)** Following viability assay, fibrogenic differentiation was analysed using ICW. **(C)** The final selected drugs were analysed for viability assay when adipogenic differentiation was analysed. RFU, Relative fluorescence units. **(D)** Following viability assay, adipogenic differentiation was analysed using ICW. Data is represented as the mean of three replicates ± standard error of the mean. Results were statistically analysed using one-way ANOVA, followed by Tukey post hoc test. Statistical significance was set at P < 0.05. **P < 0.01; ***P < 0.001.
