# Supplementary figures and images for: Strategy for drug repurposing in fibroadipogenic replacement during muscle wasting: application to duchenne muscular dystrophy
Source: Front Cell Dev Biol. 2025 Mar 26;13:1505697. doi: 10.3389/fcell.2025.1505697 (PMC11979640; doi:10.3389/fcell.2025.1505697)

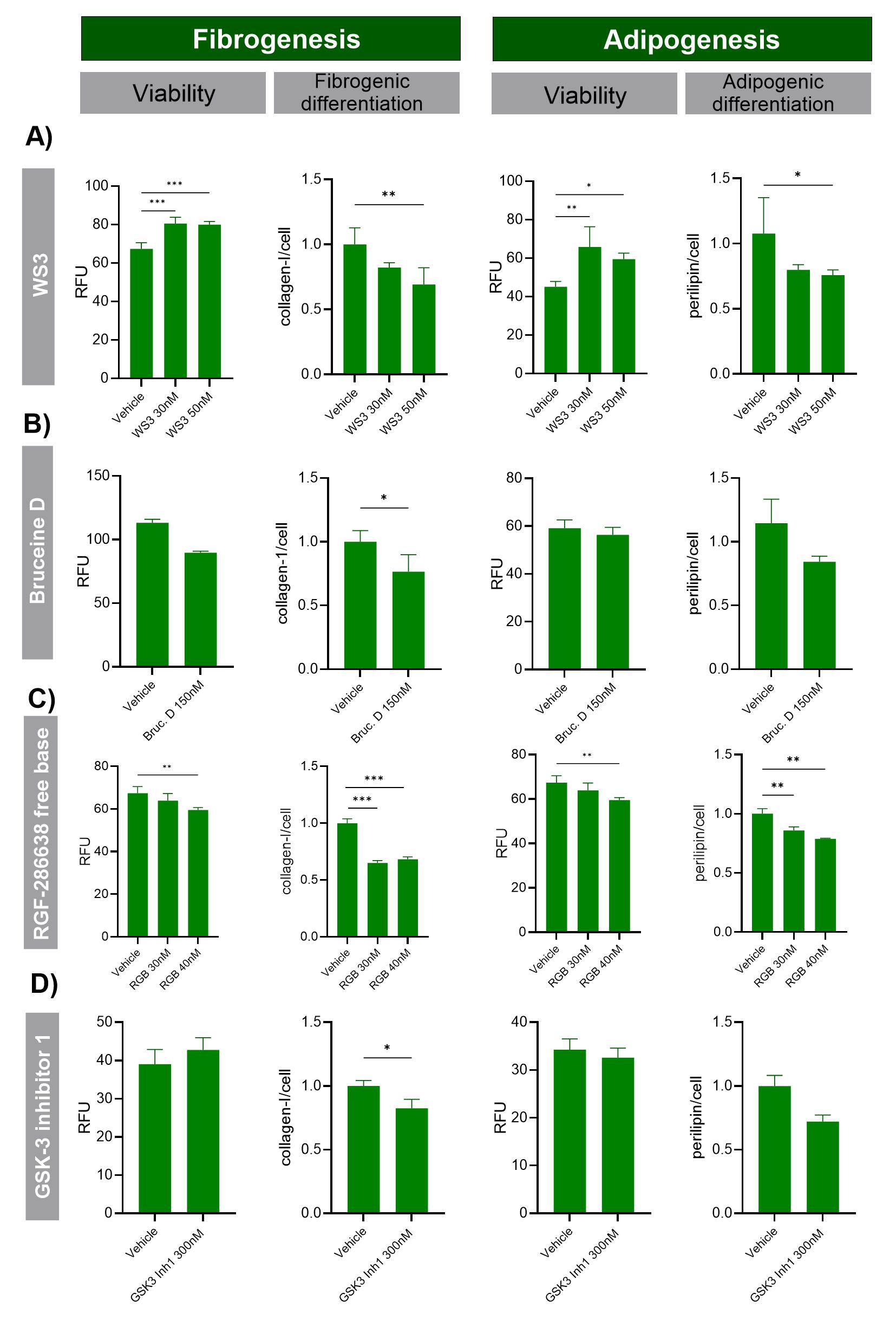

Supplement: Supplementary file 1 [file Image1.jpeg]
